# Obstructive sleep apnea is associated with impaired insulin clearance and hepatic insulin sensitivity in paediatric obesity

**DOI:** 10.1111/dom.16464

**Published:** 2025-05-14

**Authors:** Giuseppina Rosaria Umano, Francesca Aiello, Giulia Rondinelli, Alessandra Salvatori, Delfina Petrone, Maria Vittoria Sibillo, Raffaella D'Ausilio, Pierluigi Marzuillo, Anna Grandone, Grazia Cirillo, Domenico Tricò, Emanuele Miraglia del Giudice

**Affiliations:** ^1^ Department of the Woman, the Child, and General and Specialized Surgery University of Campania Luigi Vanvitelli Naples Italy; ^2^ Department of Clinical and Experimental Medicine University of Pisa Pisa Italy

**Keywords:** insulin clearance, obesity, obstructive sleep apnea

## Abstract

**Aims:**

Obstructive sleep apnea (OSA) affects up to 40% of children and adolescents with obesity and is linked to hyperinsulinism. However, the mechanisms underpinning this association remain unclear. The study aims to assess the three key determinants of hyperinsulinemia: insulin clearance, secretion, and sensitivity in paediatric patients with OSA.

**Methods:**

We enrolled 70 Children with obesity and suspected OSA who performed a nocturnal polygraphy to confirm OSA diagnosis and a 3‐hour OGTT to assess glucose homeostasis. Mild OSA was defined for 1 < AHI ≤ 5, moderate–severe OSA as AHI >5. Insulin secretion rate (ISR) was estimated using C‐peptide deconvolution. Basal and total insulin clearance during OGTT were calculated. Whole‐body insulin sensitivity was evaluated with the Matsuda Index (WBISI), while hepatic insulin resistance index (HIRI) was calculated based on the AUCs of plasma glucose and insulin during the initial 30 minutes of OGTT.

**Results:**

A total of 36 children had mild OSA and 34 had moderate–severe OSA. The latter group showed reduced insulin clearance during OGTT (*p* = 0.008) and higher HIRI (*p* = 0.03). Basal insulin clearance (*p* = 0.07), ISR (*p* = 0.34), beta‐cell glucose sensitivity (*p* = 0.53), and WBISI (*p* = 0.56) were similar between the two groups. OSA severity negatively correlated with fasting insulin clearance (*r* = −0.29, *p* = 0.01) and OGTT insulin clearance (*r* = −0.32, *p* = 0.007) and positively correlated with HIRI (*r* = 0.29, *p* = 0.02).

**Conclusion:**

Moderate–severe OSA in children with obesity is associated with impaired insulin clearance and hepatic insulin sensitivity. These factors may contribute to hyperinsulinism in paediatric OSA.

## 1 INTRODUCTION

Obstructive sleep apnea (OSA) is the most common sleep‐related breathing disorder in children.^1^ OSA is characterized by a decrease or complete halt in airflow despite an ongoing effort to breathe.^1^ Overweight and obesity increase 4–5‐fold OSA risk in children and adolescents in comparison with lean peers, and it is directly related to obesity severity.^2^, ^3^, ^4^ The estimated prevalence ranges widely between 20 and 44.6% according to different diagnostic criteria for childhood obesity and OSA.^5^, ^6^, ^7^

Obesity and OSA show a tight bidirectional causal relationship: on one side, obesity could cause an alteration of sleep pattern and quality^8^, ^9^, ^10^; on the other, strong evidence has built up that OSA per se has a negative repercussion on metabolic control including chronic hyperinsulinemia and worsening of insulin resistance, hyperglycemia, and hepatic steatosis.^11^, ^12^, ^13^, ^14^ Hyperinsulinemia has been associated with ectopic fat deposition, insulin receptor downregulation, and increased risk of dysglycemia.^15^ Several mechanisms contribute to dysglycemia in OSA subjects. The chronic intermittent hypoxia, typically found in OSA, leads to pancreatic β‐cell dysfunction and insulin resistance.^16^, ^17^, ^18^ Sympathetic activation combined with an increased corticotropic axis boosts hepatic gluconeogenesis and reduces glucose uptake from target tissues, as demonstrated in hypoxia‐exposed mice.^19^, ^20^ Furthermore, subjects with OSA present an altered inflammatory mediator pattern, leading to chronic systemic inflammation that impairs glucose homeostasis.^21^, ^22^ Although there are multiple related pathways, there is scarce clinical evidence of the pathophysiologic mechanisms underpinning the association between OSA and glucose homeostasis, especially in the paediatric population.

In a previous study, we demonstrated that OSA might reduce both static and dynamic insulin secretion without affecting whole‐body insulin sensitivity,^23^ which would reduce rather than increase plasma insulin levels. Therefore, the mechanisms underpinning hyperinsulinism in children with OSA should be clarified.

The process by which insulin is removed from circulation, named insulin clearance, is a key factor in regulating insulin levels and overall glucose homeostasis. The liver is responsible for clearing approximately 50%–60% of endogenous insulin during its first pass, but this amount is significantly reduced in youths with obesity and hepatic insulin resistance.^24^ Therefore, impaired insulin clearance in obesity has been proposed as a plausible mechanism of chronic hyperinsulinemia.^25^ Based on this knowledge, this study aims to investigate the association between OSA and insulin clearance and hepatic insulin resistance in children and adolescents with obesity.

## 2 METHODS

We enrolled children and adolescents with obesity from the outpatient obesity clinic of the University of Campania “Luigi Vanvitelli”. Children were eligible if they had a body mass index (BMI) ≥ 95th for age and sex according to reference charts^26^ with a positive screening for OSA based on the Paediatric Sleep Questionnaire.^27^

The following exclusion criteria have been applied: the use of medications affecting glucose or lipid metabolism, syndromic patients, and endocrinopathies. Written informed consent was obtained from parents, and assent was obtained from the children before any procedures were conducted. The study adhered to the guidelines of the Declaration of Helsinki and received approval from the Institutional Review Board of the University of Campania Luigi Vanvitelli (protocol no. 10364/2021). All participants underwent an anthropometric evaluation, overnight cardiorespiratory polygraphy, a 3‐hour oral glucose tolerance test (OGTT), and hepatic steatosis assessment with ultrasound, as previously reported.^23^, ^28^ OSA severity was defined according to the apnea‐hypopnea index (AHI). An AHI >1 indicated obstructive sleep apnea (OSA). Mild OSA was defined for 1 < AHI ≤5, moderate OSA as 5 < AHI < 10, and severe OSA as AHI ≥10.^23^ After an overnight fast, a blood sample was collected at 8:00 AM. The serum was then frozen at −20°C until it was analysed. An enzymatic colorimetric test with a lipid clearing factor was used to determine triglycerides, total cholesterol, LDL‐cholesterol, and HDL‐cholesterol. Serum ALT and AST levels were measured with a Hitachi Analyser.

The detection of hepatic parenchyma high‐intense defined the presence of steatotic liver disease (SLD) echoes and liver– kidney differences in echo amplitude.^28^

### 2.1 Derived OGTT‐parameters

Following a 12‐h overnight fast, an intravenous catheter was inserted into the antecubital vein after applying anaesthetic cream locally for blood sampling. Flavoured glucose at a dose of 1.75 g/kg body weight, up to a maximum of 75 g, was administered orally. Blood samples were collected every 10 min for the first 30 min after oral glucose. Subsequently, blood samples were collected every 30 minutes for 150 min. Glucose, insulin, and c‐peptide serum levels were assessed. Whole‐body insulin sensitivity was estimated by the Matsuda index.^29^

The ISR was assessed by C‐peptide deconvolution.^30^ The C‐peptide deconvolution method allows the estimation of ISR with a two‐compartment mathematical model to account for C‐peptide distribution and degradation, standardizing its kinetics for anthropometric data (weight and age) and diabetes status. Beta‐cell glucose sensitivity, describing the relationship between plasma glucose and ISR during the OGTT, was calculated using the mathematical model developed by Mari and Ferrannini.^31^

Fasting endogenous insulin clearance (CL_0_) was determined by the ratio of the fasting ISR to plasma insulin levels (ISR fast/I fast). Post‐load clearance (CL_180_) was estimated by the ratio of the AUC of ISR to the plasma insulin AUC during the 3‐hour OGTT (ISR_AUC_/*I*_AUC_).^32^, ^33^

The hepatic insulin resistance index (HIRI) was calculated to identify the hepatic component of insulin sensitivity based on OGTT plasma insulin and glucose measurements. HIRI accounts for the early (30‐minute) glucose and insulin response to an oral load. HIRI was determined as the product of the area under the curve (AUC) of plasma glucose and plasma insulin during the first 30 min of the OGTT (glucose AUC0‐30 × insulin AUC0‐30).^34^

## 3 STATISTICAL ANALYSIS

The Kolmogorov–Smirnov test was performed to assess the normal distribution of continuous variables. Results are shown as mean ± SD or median (interquartile range, IQR) or frequencies as appropriate. Differences for continuous variables were assessed with the Student *T*‐test for independent samples or the Mann–Whitney *U* test according to distribution. Fisher exact test or Chi‐square test was performed for differences in categorical variables. Differences for glucose, insulin, and c‐peptide levels during OGTT were performed by point‐by‐point comparison with a non‐parametric test because of the small sample size. Spearman correlation test was conducted to investigate the correlation between respiratory sleep parameters and OGTT‐derived parameters. All the analyses were performed using SAS® on Demand for Academics (SAS Institute Inc., Cary, NC) and Prism 8.0 (GraphPad Software, San Diego, CA).

## 4 RESULTS

**FIGURE 1:**
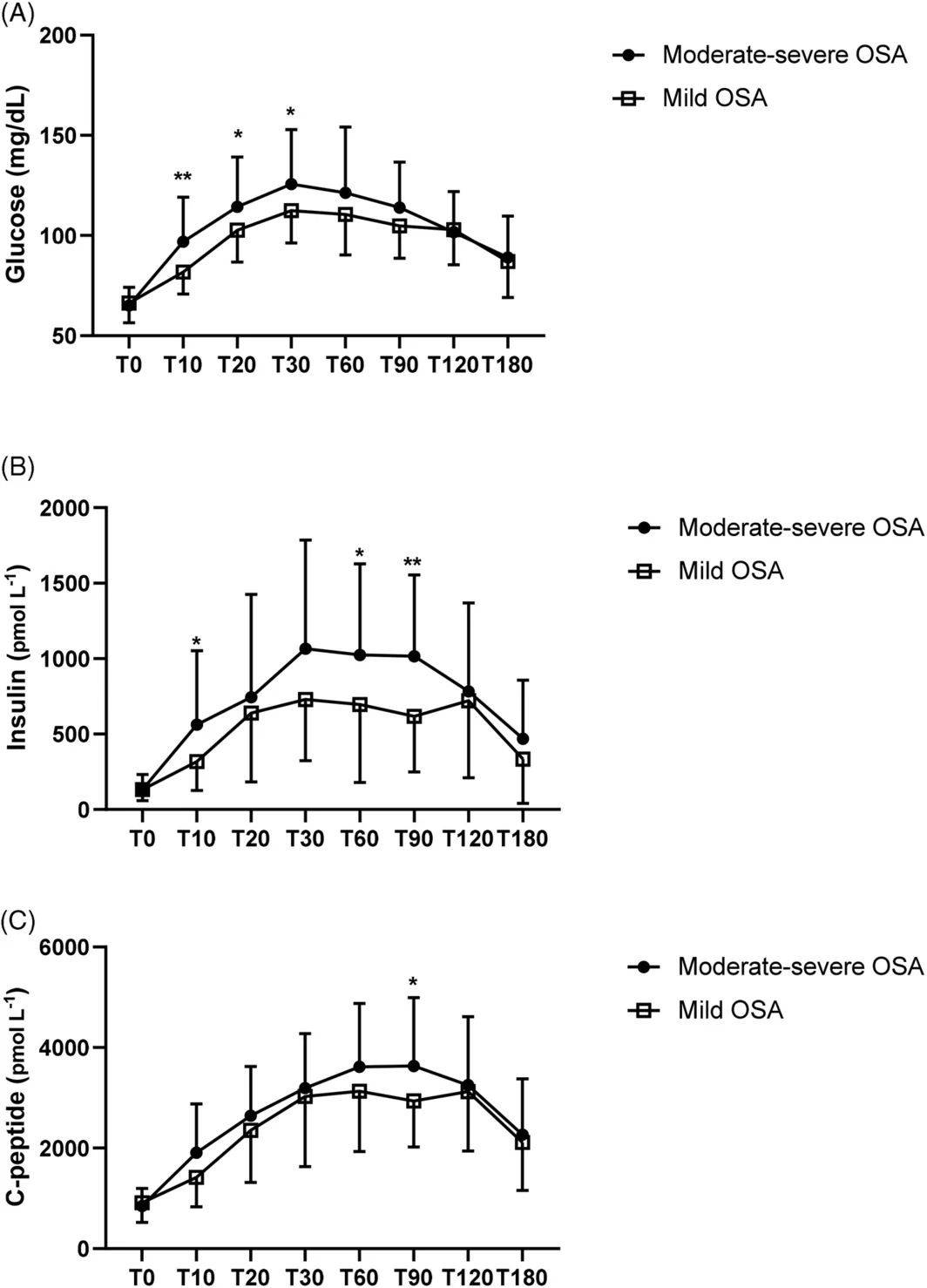
Changes in glucose, insulin, and c‐peptide levels after oral glucose by group. (A) Changes in glucose plasma levels after oral glucose by time and group. (B) Changes in insulin plasma levels by time and group. (C) Changes in c‐peptide plasma levels by time and group. Data are presented as median and interquartile range. White square refers to mild OSA group, black circle refers to moderate–severe OSA group. Stars describe statistically significant differences between groups; *: *p* < 0.05; **: *p* < 0.01.

**FIGURE 2:**
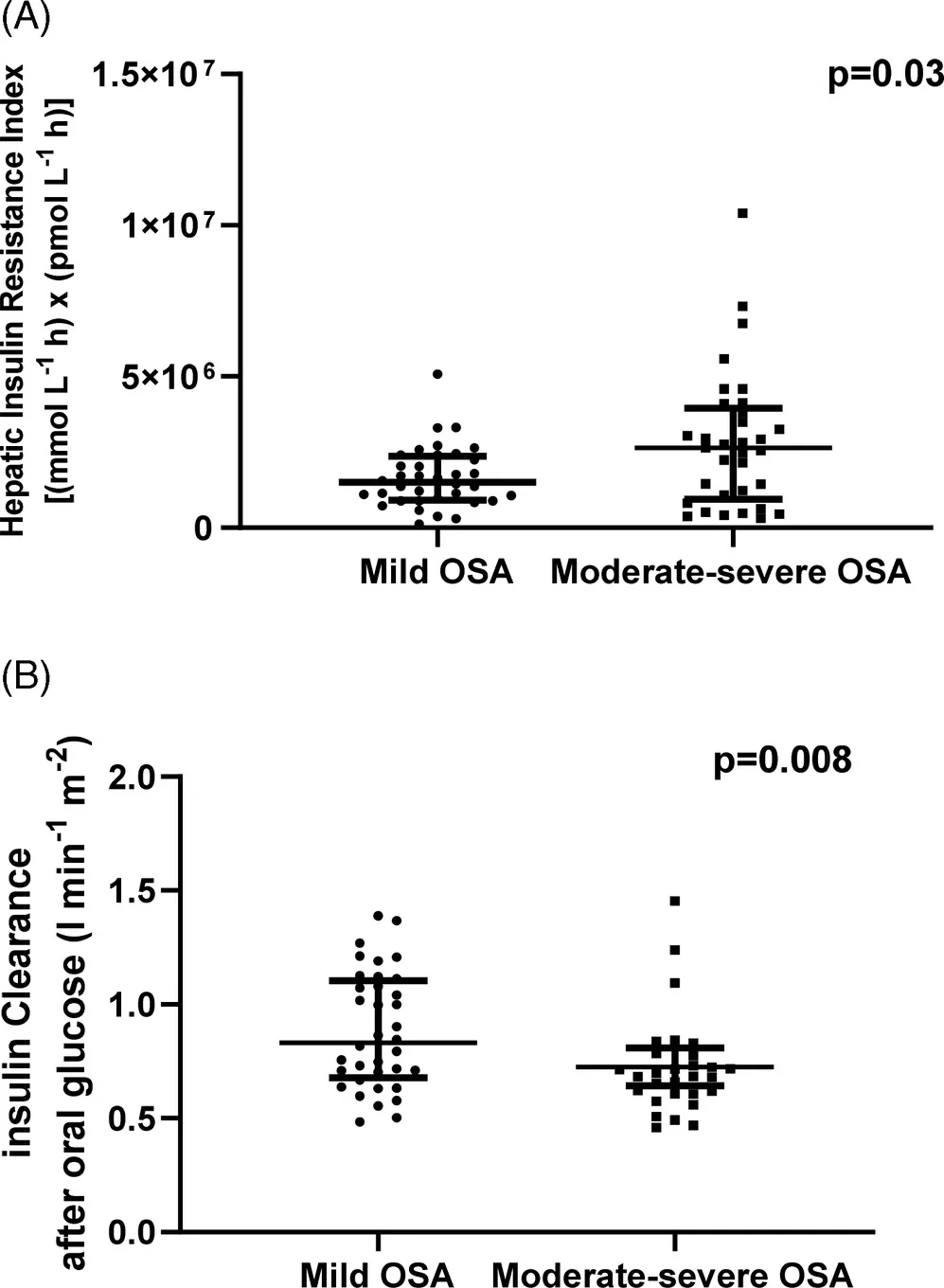
Differences in hepatic insulin resistance and insulin clearance according to group. (A) Differences in hepatic insulin resistance index by group. (B) Differences in insulin clearance by group. Black dots refer to the mild OSA group, black squares refer to the moderate–severe OSA group. Data are expressed as median and interquartile range.

**FIGURE 3:**
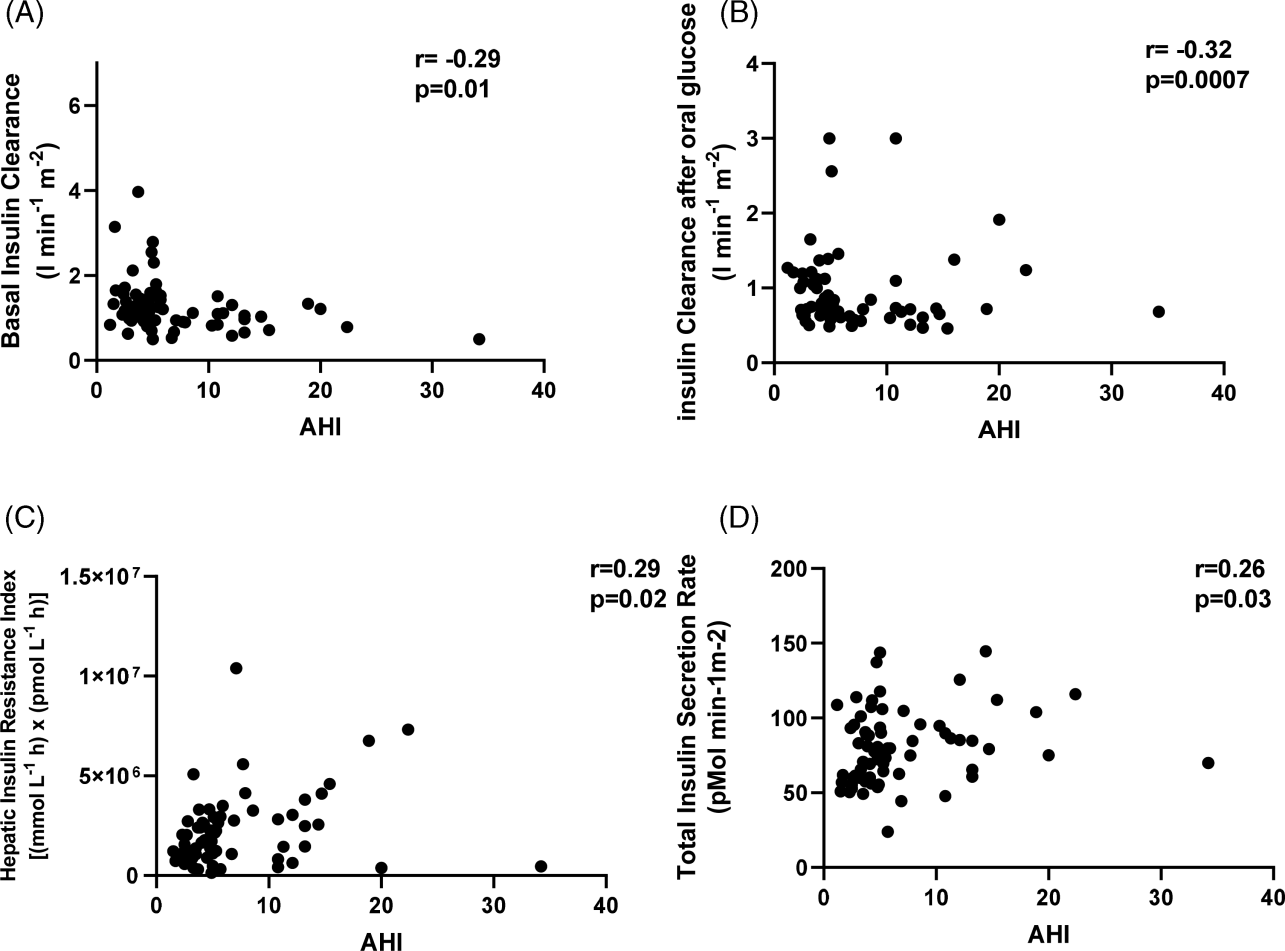
Spearman correlation between OSA severity and measures of insulin clearance, insulin secretion, and hepatic insulin resistance. (A) Correlation between AHI and basal insulin clearance. (B) Correlation between AHI and insulin clearance after oral glucose. (C) Correlation between AHI and Hepatic insulin resistance. (D) Correlation between AHI and total insulin secretion rate.

Seventy children were included in the study. Based on OSA severity, the cohort was divided into two groups (36 mild and 34 moderate–severe OSA). The two groups did not differ in age, gender, Tanner stage, weight, height, BMI, BMI z‐score, and waist‐to‐height ratio distribution (Table 1). Fasting biochemical analyses showed lower triglyceride levels in the group with mild OSA (*p* = 0.03, Table 2). Conversely, no differences were observed for glucose levels at fasting and after OGTT, measures of insulin resistance, liver enzymes, and SLD prevalence (Table 2). During the 3‐hour OGTT, the group with mild OSA displayed significantly lower glucose levels at t10, t20, and t30 minutes after oral glucose (*p* = 0.0009, *p* = 0.04, and *p* = 0.03, respectively, Figure 1A). In addition, insulin levels were significantly higher in the moderate–severe OSA group at t10, t60, and t90 minutes compared to mild OSA (*p* = 0.048, *p* = 0.02, *p* = 0.002, respectively; Figure 1B). No differences in C‐peptide levels except for t90 (*p* = 0.03, Figure 1C) were observed. Moreover, the group with moderate–severe OSA showed higher HIRI (*p* = 0.03, Figure 2A) and lower insulin clearance after oral glucose (*p* = 0.008) (Figure 2B). We observed a trend for lower insulin clearance during fasting in children and adolescents with moderate–severe OSA (*p* = 0.07). No differences were observed for basal ISR (*p* = 0.58), total ISR (*p* = 0.32), insulin sensitivity, and glucose sensitivity (*p* = 0.53) (see Table 2). OSA severity measured by the AHI was inversely correlated with basal insulin clearance (*r* = −0.29, *p* = 0.01) and insulin clearance during OGTT (*r* = −0.32, *p* = 0.007) (Figure 3). Conversely, AHI was positively correlated with HIRI (*r* = 0.29, *p* = 0.02) and total ISR (*r* = 0.26, *p* = 0.03) (Figure 3).

**TABLE 1:** Anthropometric characteristics of the cohort according to OSAS severity.

	Mild OSA (*n* = 36)	Moderate–severe OSA (*n* = 34)	*p*
Age	11.84 ± 2.65	11.53 ± 2.95	0.57
Sex (M, *n* (%))	20 (55.9)	11 (33.3)	0.09
Tanner stage (I/II‐III/IV‐V, *n* (%))	11 (31.8)/18 (50.0)/7 (18.2)	14 (40.9)/12 (36.4)/8 (24.7)	0.76
Weight (kg)	82.3 (67.1–108.5)	81.3 (63.2–95.6)	0.44
Height (cm)	156.0 (147.9–163.6)	152.0 (139.0–160.4)	0.24
BMI	35.1 (32.2–37.9)	33.9 (30.2–42.0)	0.62
Z‐score BMI	3.73 (3.20–3.92)	3.50 (3.21–3.84)	0.46
Waist‐to‐Height ratio	0.68 (0.62–0.75)	0.68 (0.63–0.72)	0.60

*Note*: Data are displayed as mean ± standard deviations or median (interquartile range) according to parameters' distribution and as number (percentages, %).

Abbreviations: BMI, body mass index; OSA, obstructive sleep apnea.

**TABLE 2:** Biochemical and instrumental characteristics of the cohort according to OSAS severity.

	Mild OSA (*n* = 36)	Moderate–severe OSA (*n* = 34)	*p*
Fasting glucose (mg/dL)	66.36 ± 9.89	65.09 ± 9.12	0.64
Fasting insulin (pmol/L)	105.0 (66.6–136.8)	90.9 (58.2–163.5)	0.58
Fasting C‐peptide (pmol/L)	2.75 (2.03–3.05)	2.47 (1.85–3.33)	0.73
HOMA‐IR	2.93 (1.67–3.75)	2.43 (1.50–4.1)	0.35
2‐h Glucose	104.0 (94.0–117.0)	99.0 (88.0–113.0)	0.25
Matsuda index (mg/dL)	3.46 (2.60–6.10)	3.53 (2.89–5.14)	0.65
Stumvoll index (mL min^−1^ kg^−1^)	4.44 ± 3.16	4.09 ± 3.30	0.79
Basal insulin clearance (L min^−1^ m^−2^)	1.23 (0.82–1.50)	1.05 (0.82–1.33)	0.07
Total insulin secretion rate (pmol min^−1^ m^−2^)	76.01 ± 21.92	86.45 ± 26.07	0.32
Glucose sensitivity (pmol min^−1^ m^−2^ mM^−1^)	127.07 (94.40–167.54)	136.83 (101.11–192.21)	0.53
Total cholesterol (mg/dL)	152.5 ± 23.55	162.8 ± 30.38	0.17
HDL‐Cholesterol (mg/dL)	39.91 ± 8.88	38.41 ± 9.10	0.79
Triglycerides	99.78 ± 37.88	124.20 ± 55.76	**0.03**
AST (UI/L)	25 (20–34)	25 (21–32)	0.66
ALT (UI/L)	24 (19–49)	28 (21–52)	0.46
SLD (%)	75.0	78.8	0.99

*Note*: Data are displayed as mean ± standard deviations or median (interquartile range) according to parameters' distribution and as percentages (%).

Abbreviations: HOMA‐IR, homeostasis model assessment of insulin resistance; SLD, steatotic liver disease.

## 5 DISCUSSION

In this study, we show that children and adolescents with obesity and moderate–severe OSA have lower insulin clearance and higher hepatic insulin resistance, but similar insulin secretion, compared to children with mild OSA. These findings support the role of impaired insulin clearance in the hyperinsulinism typically observed in youths with OSA and are in line with previous studies that reported an inverse association between insulin clearance and hepatic insulin resistance.^24^, ^35^

This is the first study that evaluates insulin clearance and hepatic insulin resistance in the context of obesity and OSA in paediatric age.

Insulin clearance, primarily performed by the liver (50–80%), is essential for managing glucose metabolism and preventing hyperinsulinemia in insulin‐resistant individuals.^36^ After pancreatic release, insulin reaches the portal vein and undergoes a first‐pass hepatic extraction.^35^ Current theories suggest this process might mitigate the negative consequences of prolonged high insulin levels, attributing a key role to the liver in regulating insulin plasma levels.^35^, ^36^ Therefore, impaired insulin clearance leads to increased insulin plasma levels and is an independent risk factor for type 2 diabetes occurrence.^37^

The underlying mechanisms of the association between obstructive sleep apnea (OSA) and reduced insulin clearance remain not completely understood. Based on pre‐clinical studies, we hypothesized several concurring mechanisms. A possible explanation links chronic intermittent hypoxia (CIH) to reduced insulin receptor internalization through impaired endosome function. CIH increases HIF1‐α expression,^38^ which promotes alternative splicing of carcinoembryonic antigen‐related cell adhesion molecule 1 (CEACAM1) with increased production of the short CEACAM1 instead of the long isoform affecting the insulin receptor turnover.^39^, ^40^, ^41^

Moreover, OSA itself can worsen SLD and further reduce insulin clearance. Current theory suggests fatty acid buildup in the liver and reduced CEACAM1 activity^42^ with lower Insulin Degrading Enzyme (IDE) levels.^43^ IDE exerts an important role in initial insulin degradation, early endosomes and microtubule polymerization, and trafficking of late endosomes (with their cargo) to recycle back the IR to the membrane during exocytosis.^44^ For the sake of completeness, CIH and IDE expression do not seem directly related, as demonstrated by Fernandés and colleagues in rats.^38^

However, we did not observe differences in SLD prevalence between the two groups. This might be related to the diagnosis of SLD being based on ultrasound rather than MRI and to the small sample size.

Besides, we can hypothesize that other mechanisms beyond SLD might link OSA and insulin resistance. Current evidence suggests intermittent hypoxia (IH) alters gut microbiota (GM), increasing gut permeability and disrupting plasma exosome cargo. These changes impair adipocyte insulin sensitivity, leading to insulin resistance.^45^ Furthermore, studies on microbial transfer from diet‐induced obese mice demonstrated that gut microbiome alteration impairs insulin clearance without altering the kinetics of the C‐peptide levels, indicating that the microbiota induced a direct effect on insulin levels rather than stimulating insulin secretion.^46^ Moreover, mouse models showed that hypoxia is associated with gut microbiome modifications and increased gut permeability to several bacterial metabolites, eventually leading to inflammation, worsening liver function and insulin sensitivity.^46^

Chronic hypoxia is responsible for sleep fragmentation with a consequent sympathetic autonomic nervous system activation.^47^ Increased sympathetic activity induces hepatic gluconeogenesis and adipose tissue lipolysis,^48^ leading to hyperglycemia and insulin resistance.^48^ Therefore, hypoxia in OSA has a key role in metabolic derangement, hampering the systemic inflammation in cases of obesity.^47^

In this cohort, we did not observe differences in whole‐body insulin sensitivity between groups. Previous studies reported contrasting results about the changes in whole‐body insulin sensitivity in OSA.^48^, ^49^, ^50^ Mouse models showed that sympathetic nervous activity and CIH were responsible for increased hepatic insulin resistance without effect on whole‐body insulin sensitivity.^48^ Conversely, Iiyori et al. reported that CIH influences glucose uptake in skeletal muscle, impairing systemic insulin sensitivity.^49^ In adults with OSA and obesity, AHI was inversely correlated with adipose tissue insulin sensitivity, without association with whole‐body insulin sensitivity assessed by the Matsuda index.^50^ However, no potential underlying mechanisms were investigated to explain these observations.^50^ Considering the relatively small sample size and the possible selection bias linked to a selected population in this study, our findings cannot disambiguate the contrasting literature.

In a previous study, we reported that paediatric OSA was associated with impaired static and dynamic insulin secretion without differences in insulin resistance.^23^ In this study, we observed higher insulin levels during OGTT in moderate–severe OSA without differences in C‐peptide levels between the two groups. Therefore, we might speculate that children and adolescents with obesity and severe OSA might display hyperinsulinism because of impaired insulin clearance instead of increased insulin secretion. The lack of differences in insulin secretion might also be due to the methods applied for insulin secretion estimation, namely the C‐peptide deconvolution method. This model, albeit validated,^29^ is not the gold standard for insulin secretion assessment.

We acknowledge that this study presents several limitations. The cross‐sectional design does not allow for dissecting the direction of the reciprocal influence between hepatic insulin resistance, impaired insulin clearance, and OSA. In addition, the lack of a control group without OSA limits the ability to distinguish the effect of OSA independently from the effect of obesity. The use of model‐derived parameters instead of the gold standard clamp method tends to be more sensitive to minor differences and might reduce the strength of the results. Moreover, the small sample size and the single‐center design might have introduced selection bias. Nonetheless, it should be recognized that these procedures (the 3‐h OGTT and nocturnal polygraphy) are challenging to perform in children. More research with longitudinal and/or intervention design with a multi‐omic approach might help in dissecting this complex interrelationship between obesity, OSA, and metabolic derangement even during childhood. Understanding the mechanisms linking OSAS to impaired insulin clearance is crucial for developing targeted preventive and therapeutic strategies, ensuring early intervention and reducing the risk of type 2 diabetes.

## AUTHOR CONTRIBUTIONS

GRU study conceptualization, manuscript writing; FA manuscript writing; GR, AS, and DP data collection; MVS and RDA sleep study; PM and AG data analysis; GC biochemical analyses; DT manuscript writing and data analysis, EMDG supervision and conceptualization.

## CONFLICT OF INTEREST STATEMENT

The authors have no competing interest to disclose.

## Data Availability

Some or all datasets generated during and/or analysed during the current study are not publicly available but are available from the corresponding author on reasonable request.
